# The dynamic locking blade plate: seven-year follow-up results of 389 patients with a femoral neck fracture

**DOI:** 10.1007/s00068-024-02552-5

**Published:** 2024-05-31

**Authors:** J. H. Kalsbeek, W. H. Roerdink, P. Krijnen, C. A.S. Berende, J. T. Winkelhorst, A. D. P. van Walsum, I. B. Schipper

**Affiliations:** 1 Department of Trauma Surgery, Medisch Spectrum Twente, Koningstraat 1, Enschede, 7512 KZ The Netherlands; 2 Department of Trauma Surgery, Leiden University Medical Center, Albinusdreef 2, Leiden, 2333 ZA The Netherlands; 3 Department of Trauma Surgery, Deventer Ziekenhuis, Nico Bolkesteinlaan 75, Deventer, 7416 SE The Netherlands; 4 Acute Care Network West Netherlands, Rijnsburgerweg 10, Leiden, 2333 AA The Netherlands; 5 Department of Trauma Surgery, Amphia Ziekenhuis, Molengracht 21, Breda, 4818 CK The Netherlands; 6 Department of Trauma Surgery, Canisius Wilhelmina Ziekenhuis, Weg door Jonkerbos 100, Nijmegen, 6532 SZ The Netherlands

**Keywords:** Femoral neck fracture, Intracapsular hip fracture, Hip fracture, Displaced undisplaced, Dynamic locking blade plate, Internal fixation

## Abstract

**Purpose:**

This study aimed to investigate the long-term outcomes of patients with a femoral neck fracture (FNF), treated with the Dynamic Locking Blade Plate (DLBP).

**Methods:**

Retrospective analysis of prospectively collected data of a multicentre cohort of patients with FNFs was conducted, regarding the long-term incidence of revision surgery after DLBP. Implant failure was evaluated using Kaplan-Meier and Cox regression analysis. Secondary outcomes were the indication for revision surgery, complications, time to revision surgery, rate of elective removal of the implant, potential predictors for revision surgery and mortality.

**Results:**

Median follow-up of 389 included patients was 98 months; 20.6% underwent revision surgery; 28.8% after treatment of a displaced FNF (dFNF) and 10.0% with a undisplaced FNF (uFNF). 5.7% (*n* = 22) of the patients had operation related complications and 32.9% (*n* = 128) deceased during follow-up. Median time to revision surgery was 13 (dFNF) and 18 months (uFNF). 15.7% of the DLBPs were electively removed. In the multivariate Cox regression analysis, female gender (hazard ratio 2.1, 95% CI 1.2–3.7) and a TAD > 25 mm (hazard ratio 2.9, 95% CI 1.7-5) were significant predictors for revision surgery in patients with dFNF.

**Conclusion:**

This study is the first long-term follow-up study on the outcome of the DLBP. The DLBP demonstrated positive long-term results in the treatment of FNF.

**Supplementary Information:**

The online version contains supplementary material available at 10.1007/s00068-024-02552-5.

## Introduction

The treatment of femoral neck fractures (FNFs) has been discussed extensively for many decades. The two main surgical treatment options are internal fixation and (hemi)arthroplasty. However, both treatments know potential disadvantages and complications. Arthroplasty of the hip is associated with dislocation, pulmonary embolism, cement induced syndrome, limited implant survival and severe morbidity due to infection [1, 2], Internal fixation is associated with high failure rates (10–49%) due to non-union, avascular necrosis (AVN) of the femoral head and cut-out of the implant [3].

To improve the outcome of head preserving treatment of FNFs, the Dynamic Locking Blade Plate (DLBP) was developed, a fixed angle device characterized by its excellent rotation stability, low implant volume and simple operation technique [4–6]. The DLBP demonstrated good results in treatment of FNF, with 4% failure rate at one year in a prospective multicentre cohort study of 172 patients with undisplaced FNFs (uFNF) [5]. Another prospective cohort study of 106 young patients with displaced FNFs (dFNF) demonstrated a failure rate of 13.2% after one year [6]. A randomised controlled trial comparing the DLBP with the Dynamic Hip Screw is ongoing at this moment [7].

The follow-up of these studies was limited because most of the complication occur within one year. Yet some FNFs are still revised after one year [8, 9]. Kelly et al. found a mean time to revision of 1.3 years (range 73 days– 4.9 years) within a cohort of FNFs treated with a sliding hip screw with a mean follow-up time of 8.2 years (range 6.7–10.1) [10]. Mean time to AVN varies between 16 and 18.8 months with a total range of 3–60 months [11–13]. Patients may also suffer from posttraumatic osteoarthritis (PTOA) which can present years after the initial trauma. In 35% of patients treated for FNF, PTOA is found to be the indication for subsequent total hip arthroplasty [14].

The aim of this study was to investigate the long-term outcomes of patients with an FNF, treated with the DLBP. More specifically, the need for and type of revision surgery were evaluated, as were complication and mortality rates in patients with a minimum of seven years follow-up.

## Patients and methods

### Design and cohort

Retrospective analysis of prospectively documented data of a multicentre cohort including 468 adult patients treated with the DLBP between 2010 and 2014 was conducted. In this cohort all consecutive patients of any age with a uFNF, patients with a dFNF of ≤ 65 years and patients between 65 and 75 years who were not dependent on walking aids and who were not admitted in a nursing home prior to the fracture were included. Their prospectively registered data including clinical outcomes up to one year have been published previously [5, 6]. For the present study, all patients with an FNF from this cohort with a minimum follow-up of seven years, or shorter if the endpoint (revision surgery or death) was reached before seven years of follow up, were eligible. Patients who met any of the following criteria were excluded in this study:

- Pathological fracture.
- Ipsilateral or contralateral fracture(s) of the lower extremity.
- Symptomatic osteoarthritis or radiographic osteoarthritis grade III or IV [15].
- Previous surgery of the ipsilateral hip.
- Patients who were wheelchair-bound in their pre-injury situation.
- Patients who were not mentally competent to take a survey.

### Study outcome parameters

The main study outcome parameter was the incidence of revision surgery, defined as any reoperation due to failure of treatment (cut-out of the implant, AVN, non-union, or PTOA), such as conversion to (total) hip arthroplasty, girdle stone, core decompression, vascular fibular graft, valgus (intertrochanteric) osteotomy. Elective removal of the DLBP after union of the fracture was not considered as revision surgery.

Secondary outcome parameters were the indications for revision surgery (AVN, non-union, cut-out of the implant, PTOA), index-operation related complications such as bleeding or infection, time to revision surgery, elective removal of the DLBP, and mortality. AVN was defined as stage 2 necrosis and upward, according to the Steinberg classification [16]. Our definition of non-union was based on the Radiographic Union Score for Hip (RUSH) [17]. Non-union was defined as a visible fracture line on the radiograph, absence of cortical bridging or bridging trabeculae over the fracture site in combination with persisting pain in the hip or the inability to bear weight for at least 9 months after surgery or sooner if revision surgery was performed because it was no longer expected that fracture healing would occur. PTOA was defined as having symptoms of OA including pain, stiffness of the joint with or without radiologic findings of OA in absence of AVN, non-union or cut-out of the implant.

Other data registered in the database were age, gender, fracture displacement, posterior tilt of the femoral head, time to surgery, operation time, reduction of the fracture, Tip-Apex-Distance (TAD), postoperative complications, mortality and revision rate and indication for revision at one year. Fracture displacement was assessed according to the Garden classification with uFNF defined as Garden type 1 & 2 and dFNF as Garden type 3 & 4 [18]. The Garden Alignment Index was used to evaluate the fracture reduction on the first postoperative radiograph [19]. The acceptable range of reduction is a 160 to 180° angle [20, 21]. Posterior tilt was measured using the posterior tilt measurement according to Palm et al. [22].

### Study procedure

If patients were eligible for the present study, outcome data additional to their one-year follow-up data were collected from the medical records. If information on the presence or absence of revision surgery during at least seven years, and if applicable peri-operative information of the revision surgery, could not be found in the records, the patient was contacted by telephone. If information on the primary outcome could not be retrieved the patient was excluded. Seven years follow-up was chosen because time between implantation of a DLBP in the last included patient and the start of data collection was seven years.

### Statistical analysis

Statistical analysis was performed using SPSS Statistics 28 software (IBM Corp., Armonk, New York) for Windows 10 Home (Microsoft, Redmond, Washington). The data was analysed in subgroups of patients with uFNF and dFNF. Results are presented as frequency and percentage for categorical data and mean (standard deviation) or median (range) for continuous data. Difference in revision rate between the groups with uFNF and dFNF was evaluated using Kaplan-Meier survival curves and compared with the log-rank test. Kaplan-Meier analysis was also used to compare revision surgery rates between subgroups of patients with various indications for revision surgery (AVN, non-union, cut-out of the implant, posttraumatic OA). Potential predictors for revision surgery (female gender, operation performed by a surgical resident, TAD > 25 mm, age > 65 years, inadequate reduction for dFNF and posterior tilt > 20° in uFNF were assessed using univariable and multivariable Cox proportional hazards regression analysis, separately for the uFNF and dFNF groups. Statistical significance was set at *p* < 0.05.

### Ethical approval

This study was conducted in compliance with the declaration of Helsinki (2008) and the principles of good clinical practice (GCP). According to the institutional Medical Research Ethics Committee, this study did not meet the criteria of the Dutch Medical Research Involving Human Subjects Act (WMO), so that ethical approval was not needed.

## Results

**Fig. 1:**
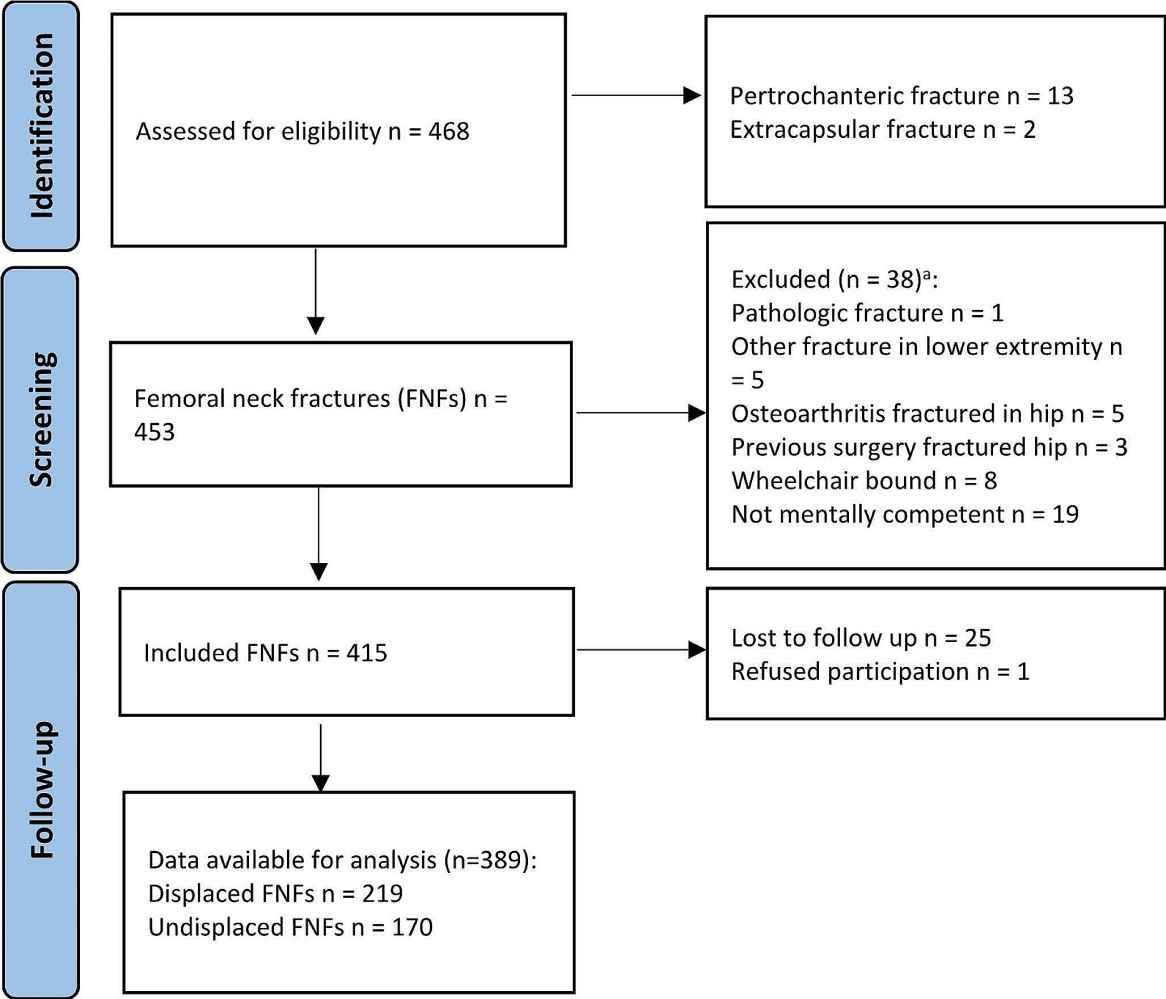
STROBE flow diagram. ^a^ Some patients met multiple exclusion criteria

Seventy-nine of the 468 patients in the cohort were excluded, leaving 389 patients eligible for analysis (219 with dFNF and 170 with uFNF). Reasons for exclusion are presented in Fig. 1. The characteristics of the study population are presented in Table 1. Median follow-up was 98 months (range 0-150) and similar in both dFNF and uFNF.

**Table 1:** Demographic and clinical characteristics of 389 included patients

Variable	Total *n* = 389	Displaced FNF *n* = 219	Undisplaced FNF *n* = 170
Mean age, in years (SD)	65.8 (13.3)	62.8 (12.2)	69.7 (13.7)
Male, n (%)	169 (43.4)	106 (48.4)	63 (37.1)
Mean operation time, in minutes (SD)^a^	43.1 (18.7)	43.9 (19.5)	42.0 (17.7)
Operating physician, n (%)^b^			
Surgeon	328 (84.8)	183 (83.6)	145 (86.3)
Surgical resident	59 (15.2)	36 (16.4)	23 (13.7)
Time to surgery, n (%)			
within 24 h	321 (82.9)	187 (85.8)	134 (79.3)
within 48 h	362 (93.5)	206 (94.5)	156 (92.3)
Mean TAD, in mm (SD)^c^	21.4 (6.9)	21.6 (7.2)	20.1(6.5)
Mean posterior tilt, in degrees (SD)	13.8 (9.8)	N/A	13.8 (9.8)
Inadequate reduction, n (%)	48 (12.9)	29 (13.8)	19 (11.8)
Median follow-up, in months (range)	98 (0-150)	99 (0-150)	98 (0-150)

^a^*N* = 9 missing

^b^*N* = 2 missing

^c^*N* = 21 missing

FNF = femoral neck fracture; SD = standard deviation; TAD = Tip-Apex-Distance; N/A = not applicable

**Fig. 2:**
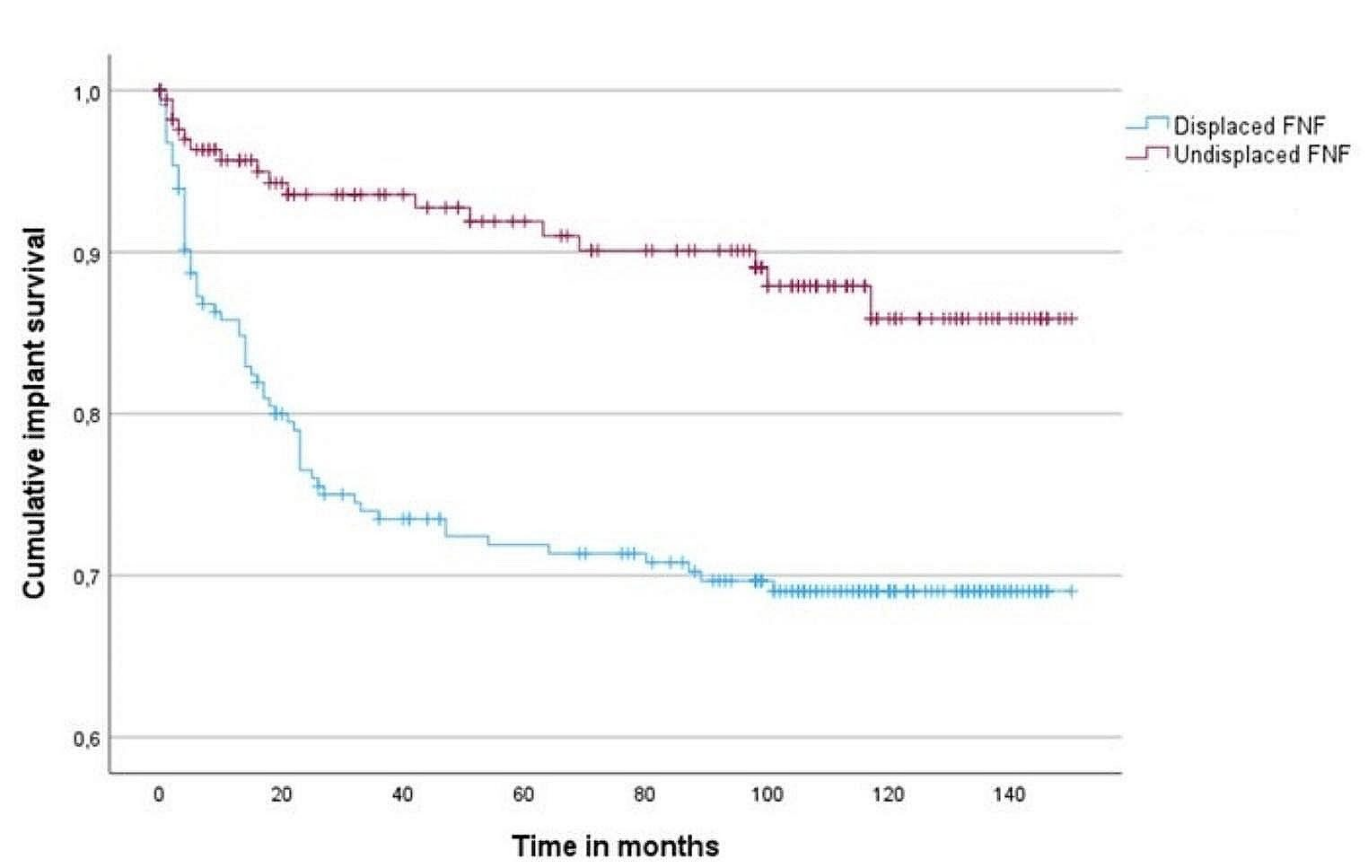
Kaplan–Meier curves for time to revision surgery in patients with displaced FNF and undisplaced FNF. Log-rank test, p = < 0.001

Of the 389 patients, 80 patients (20.6%) underwent revision surgery, 63 of the 219 patients with a dFNF (28.8%) and 17 of the 170 patients with a uFNF (10.0%). The rate of revision surgery was higher for the dFNF group than for the uFNF group (Fig. 2; log-rank test *p* < 0.001)Twenty-two patients (5.7%) had index-operation related complications(Table 2). Almost one third of all patients (*n* = 128, 32.9%) died during follow up. There were three instances of in-hospital deaths; one patient died after a cardiac event, another patient experienced a fatal pneumonia and the third patient expired due to heart failure secondary to a pneumonia.

**Table 2:** Perioperative complications in 389 femoral neck fracture patients

	*n*
Wings of implant did not expand (partially)	9
Piece of drill head remained in femoral head	1
Fracture Related Infection	3
Gauze remained in wound	1
Device malfunction	1
Postoperative bleeding	6
Blade measured too short and was revised in a second operation	1
Total	22

**Fig. 3:**
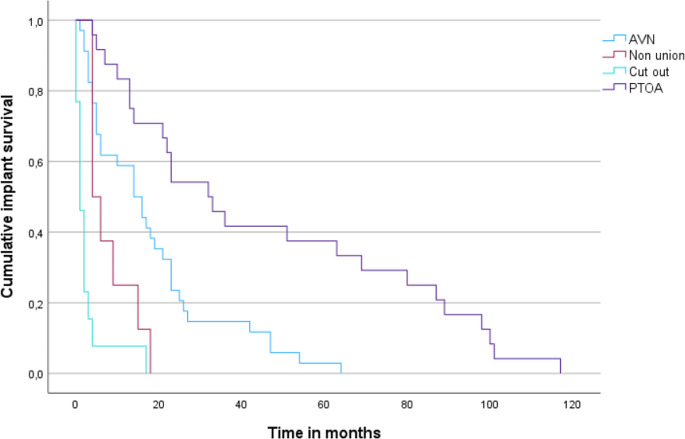
Kaplan–Meier curves for time to revision surgery by indication for revision. Log-rank *p* < 0.001. AVN = avascular necrosis, PTOA = Posttraumatic osteoarthritis

The type and indications for revision surgery for dFNF and uFNF are presented in Table 3. Although the difference in indications for revision surgery did not reach statistical significance, dFNFs seemed to be more often revised due to AVN than uFNFs (46.0% vs. 29.4% p-value = 0.22), while uFNFs seemed to more often need revision due to PTOA (47.1% vs. 25.4%, p-value = 0.08). Figure 3 shows the Kaplan Meier curve for time to revision surgery per indication for revision for dFNF and uFNF together. Median time to revision for PTOA was 32 months and respectively 1, 4 and 14 months for cut-out, non-union and AVN (log rank test, *p* < 0.001). In three patients treatment failed (two cut-out, one AVN), yet no revision was performed because the patients could not be operated; two patients had severe lung disease and one patient was 90 years old and had a poor clinical condition. Sixty-one (15.7%) DLBPs were electively removed after union.

**Table 3:** Type and indication for revision surgery after treatment with the Dynamic Locking Blade Plate

Revision type	Total *n* = 80	Displaced FNF *n* = 63	Undisplaced FNF *n* = 17	*P*-value
Total hip arthroplasty, n (%)	74 (92.5)	58 (92.1)	16 (94.1)	0.78
Hemiarthroplasty, n (%)	6 (7.5)	5 (7.9)	1 (5.9)	
**Indication** ^**a**^				
Avascular necrosis, n (%)	34 (42.5)	29 (46.0)	5 (29.4)	0.22
Non-union, n (%)	8 (10.0)	7 (11.1)	1 (5.9)	0.52
Cut out, n (%)	13 (16.3)	10 (15.9)	3 (17.6)	0.86
Posttraumatic osteoarthritis, n (%)	24 (30.0)	16 (25.4)	8 (47.1)	0.08

^a^ One patient in the displaced FNF group was revised abroad so that the indication for revision could not be determined

FNF = femoral neck fracture

The multivariable Cox proportional hazards regression analysis did not identify any predictors for revision surgery in the uFNF group (Table 4). Female gender (hazard ratio 1.99, 95% CI 1.14–3.49) and a TAD > 25 mm (hazard ratio 2.66, 95% CI 1.52–4.67) were statistically significant predictors for revision surgery in patients with dFNF. Notable was that fracture reduction was not a significant predictor for revision surgery in the dFNF group. Yet in a multivariate analysis, fracture reduction was a significant predictor for AVN after dFNF (HR 2.90, CI 95% 1.20–7.01, supplementary material 1). None of the other characteristics were predictors for AVN.

**Table 4:** Predictors for revision surgery of femoral neck fractures (FNF) treated with the Dynamic Locking Blade Plate: uni- and multi-variable Cox regression analyses

Displaced FNF		Univariable analysis		Multivariable analysis	
		*Hazard ratio (95% CI)*	*P-value*	*Hazard ratio*	*P-value*
Gender	Male	Reference			
	Female	1.70 (1.02–2.83)	0.04	1.99 (1.14–3.49)	0.02
Surgeon	Surgeon	Reference			
	Surgical resident	1.06 (0.55–2.02)	0.87	1.04 (0.53–2.04)	0.91
TAD	≤ 25 mm	Reference			
	> 25 mm	2.20 (1.31–3.69)	0.03	2.66 (1.52–4.67)	< 0.001
Age	≤ 65 years	Reference			
	66–75 years	1.50 (0.89–2.57)	0.13	1.33 (0.76–2.33)	0.32
	> 75 years	1.28 (0.56–2.90)	0.56	1.47 (0.64–3.38)	0.36
Reduction	Adequate reduction	Reference			
	Inadequate reduction	1.75 (0.93–3.29)	0.83	1.53	0.22
**Undisplaced FNF**					
Gender	Male	Reference			
	Female	2.71 (0.78–9.44)	0.12	2.79 (0.78–10.02)	0.12
Surgeon	Surgeon	Reference			
	Surgical resident	2.76 (0.97–7.87)	0.06	2.30 (0.76–6.95)	0.14
TAD	< 25 mm	Reference			
	≥ 25 mm	1.16 (0.38–3.54)	0.80	1.64 (0.51–5.23)	0.41
Age	≤ 65 years	Reference			
	66–75 years	2.08 (0.67–6.49)	0.21	1.85 (0.58–5.97)	0.30
	> 75 years	1.40 (0.42–4.68)	0.59	1.45 (0.43–4.91)	0.55
Posterior tilt	≤ 20°	Reference			
	> 20°	1.80 (0.68–4.72)	0.24	1.87 (0.70–5.01)	0.22

## Discussion

In this study we evaluated the revision rate of FNF treated with the DLBP during a minimum follow-up of seven years. The revision rate was 10.0% in uFNF and 28.8% in dFNF. Long-term results of large prospective multicentre cohorts of patients with FNF are rare in literature. There are only few studies investigating outcomes of more than 100 patients with FNFs treated with IF, with a follow-up of at least five years [23–27]. All of these studies concerned treatment with cannulated screws or pins. The rates of revision surgery presented in these studies range from similar to our results to much higher percentages, 31.3-45.6% in dFNF and 10.7-19% in uFNF. We have not been able to find any large, long-term studies of FNF treated with a DHS. However, studies with a short (i.e. 12 to 24-month) follow-up have shown revision rates of 24–41% in dFNF and 9.8-16.3% [9, 28–29]. Low implant volume and high angular and rotational stability may attribute to improved stability of the fracture-implant complex and subsequent lower revision rates. However, it also could be that these five studies included predominantly older people, with an average age of 77–82 years for patients with a dFNF and 79–81 years for patients with a uFNF, compared to 63 and 70 years respectively in our study [23–27]. These age differences render comparison of results and interpretation of findings of these studies difficult.

In concordance with earlier findings in literature, dFNFs were revised more often than uFNFs (28.8% vs. 10%). These numbers are higher than previously reported revision rates of the DLBP with a follow-up of one year [5, 6]. In our cohort the median time to revision surgery was 13 months for dFNF and 18 for uFNF. Other studies also found a median time to revision surgery of more than one year [10–13]. Figure 2 demonstrates dFNF were revised more often in the first 24 months. This might be due to the specific indications for revision. We found that 46% of the dFNFs needed revision surgery because of AVN, in comparison to 29.4% of uFNF. UFNFs were more often revised because of PTOA (25.4% vs. 47.1%). Although this difference is not significant, probably because of the low rate of revisions in the uFNF group, the results present a trend regarding the different indications for revision surgery in dFNFs and uFNFs. Figure 3 shows that AVN was revised at an earlier stage than PTOA. Median time until revision for AVN was 14 months and 32 months for PTOA. 30% of all revisions were due to PTOA. These numbers are corresponding with the 35–38% described in literature [14, 30]. However, there is no other literature describing time to PTOA and overall incidence of PTOA after FNF.

We could not find any independent predictors for revision surgery in uFNFs. This might be due to a lack of statistical power with only 17 revisions in the undisplaced fracture group of 170 patients. The inherent stability of an undisplaced fracture and the preservation of a significant portion of vascularization in these fractures will probably be the main factors that attribute to low revision rates. Female gender and a TAD > 25 mm showed to be independent predictors for revision surgery in displaced hip fractures and increased the risk respectively 2.0 and 2.7 times. A recent meta-analysis provided an overview of predictors for revision of internally fixated dFNFs and also identified female gender as a predictor for revision surgery (OR 1.78, 95% CI 1.26–2.52) [31]. TAD was not associated with a higher risk of revision surgery in this systematic review. However only one included study in this review described TAD as a possible predictor and interestingly, this study utilized the same population as the current study with one year follow-up [6]. TAD is widely described as a predictor for failure after fixated extracapsular fractures but not for intracapsular FNFs [32]. On the other hand, reduction of the fracture has been widely described in literature as a predictor for revision surgery after internal fixation of FNFs [31]. However, based on our data, fracture reduction did not emerge as a significant predictor. Fracture reduction has a large influence on the vascularisation of the femoral head and therefore on the risk of AVN. Due to the long follow-up of this study a rather large amount of patients with dFNFs were revised because of PTOA (25.4%). The follow-up in the studies included in the meta-analysis of Kalsbeek et al. was 24 months at most versus a median follow-up of 98 months in this study. Possibly these patients are mainly revised due to AVN and therefore reduction has more influence on the revision rate. We could not retrieve information on the proportion of FNFs that needed revision because of AVN in the studies included in the meta-analysis. To test the hypothesis that reduction of the fracture is a predictor for AVN we performed a multivariable Cox regression analysis on our data (supplementary material 1). Fracture reduction was a predictor for AVN in dFNF (HR 2.90, CI 95% 1.20–7.01). This indicates that reduction of the fracture is essential to ensure viable vascularisation to the femoral head and prevent AVN.

PTOA may occur due to the fact that a fractured hip, even with optimal reduction, will never fully regain its original anatomical integrity. Due to these (little) biomechanical changes the load distribution through the hip differs and accelerates decline of joint cartilage. Furthermore, a decreased vascularisation of the femoral head and cartilage after an FNF in elderly patients could impair the ability to slow this increased degeneration.

Strengths of this study are its large cohort, its prospective inclusion of the patients, long follow-up and the small percentage of patients lost to follow-up. A small part of the data was collected retrospectively. This causes limitations inherent to a retrospective set-up. Furthermore, we studied a young population with FNFs whereas most other studies will show a higher average age of their study population. This age difference renders comparison between groups difficult. Other limitations may be caused by the absence of a valid functional outcome and the lack of registration of more possible predictors for revision surgery, such as smoking [31].

## Conclusion

This study is the first long-term follow-up study on the outcome of the DLBP and first large long-term cohort study of FNFs with a relatively young population. Although study populations differ throughout literature and are not exactly comparable to our patients cohort, the DLBP demonstrated positive long-term results in the treatment of FNFs, with an overall 7-year revision rate of 20.6%, a revision rate of 28.8% for dFNF, and 10.0% for uFNF. Our study identified female gender and a TAD > 25 mm as predictors for revision in dFNF.

## Electronic supplementary material

Below is the link to the electronic supplementary material.

Supplementary Material 1

## Data Availability

Data will be available upon request.

## Code availability

Statistical analysis was performed using SPSS Statistics 28 software (IBM Corp., Armonk, New York) for Windows 10 Home (Microsoft, Redmond, Washington).

## References

[1] Burgers PT, Van Geene AR, Van den Bekerom MP, et al. Total hip arthroplasty versus hemiarthroplasty for displaced femoral neck fractures in the healthy elderly: a meta-analysis and systematic review of randomized trials. Int Orthop. 2012;36:1549–60. 22623062 10.1007/s00264-012-1569-7 PMC3535035

[2] Evans J, Evans J, Walker R, Blom A, Whitehouse M, Sayers A. How long does a hip replacement last? A systematic review and meta-analysis of case series and national registry reports with more than 15 years of follow-up. Lancet. 2019;393:647–54. 30782340 10.1016/S0140-6736(18)31665-9 PMC6376618

[3] Bhandari M, Devereaux PJ, Swiontkowski MF, et al. Internal fixation compared with arthroplasty for displaced fractures of the femoral neck. A meta-analysis. J Bone Joint Surg Am. 2003;85–A:1673–81. 10.2106/00004623-200309000-00004 12954824

[4] Roerdink WH, Aalsma AM, Nijenbanning G, van Walsum AD. Initial promising results of the dynamic locking blade plate, a new implant for the fixation of intracapsular hip fractures: results of a pilot study. Arch Orthop Trauma Surg. 2011;131:519–24. 20963430 10.1007/s00402-010-1195-z

[5] van Walsum ADP, Vroemen J, Janzing HMJ, Winkelhorst T, Kalsbeek J, Roerdink WH. Low failure rate by means of DLBP fixation of undisplaced femoral neck fractures. Eur J Trauma Emerg Surg. 2017;43:475–80. 27084541 10.1007/s00068-016-0659-4 PMC5533819

[6] Kalsbeek JH, van Walsum ADP, Vroemen JPAM, et al. Displaced femoral neck fractures in patients 60 years of age or younger: results of internal fixation with the dynamic locking blade plate. Bone Joint J. 2018;100–B:443–9. 29629591 10.1302/0301-620X.100B4.BJJ-2016-1098.R3

[7] Kalsbeek JH, Roerdink WH, Krijnen P, van den Akker-van Marle ME, Schipper IB. Study protocol for the DEFENDD trial: an RCT on the dynamic locking blade plate (DLBP) versus the dynamic hip screw (DHS) for displaced femoral neck fractures in patients 65 years and younger. BMC Musculoskelet Disord. 2020;21:139020–3131. 10.1186/s12891-020-3131-x PMC7055123 32126995

[8] Davison JN, Calder SJ, Anderson GH, et al. Treatment for displaced intracapsular fracture of the proximal femur. A prospective, randomised trial in patients aged 65 to 79 years. J Bone Joint Surg Br. 2001;83:206–12. 11284567 10.1302/0301-620x.83b2.11128

[9] Fixation using Alternative Implants for the Treatment of Hip fractures (FAITH). Investigators. Fracture fixation in the operative management of hip fractures (FAITH): an international, multicentre, randomised controlled trial. Lancet. 2017;389:1519–27. 28262269 10.1016/S0140-6736(17)30066-1 PMC5597430

[10] Kelly MA, McSorley K, Casey MC, Shannon FJ. The long-term outcomes following internal fixation for intracapsular hip fractures in an Irish tertiary referral centre. Ir J Med Sci. 2019;188:1227–31. 30712244 10.1007/s11845-019-01972-2

[11] Cho MR, Lee SW, Shin DK, et al. A predictive method for subsequent avascular necrosis of the femoral head (AVNFH) by observation of bleeding from the cannulated screw used for fixation of intracapsular femoral neck fractures. J Orthop Trauma. 2007;21:158–64. 17473751 10.1097/BOT.0b013e31803773ae

[12] Min BW, Kim SJ. Avascular necrosis of the femoral head after osteosynthesis of femoral neck fracture. Orthopedics. 2011;34:34920110317–13. 10.3928/01477447-20110317-13 21598895

[13] Ju F, Hou R, Xiong J, Shi H, Chen Y, Wang J. Outcomes of femoral Neck fractures treated with Cannulated Internal Fixation in Elderly patients: a long-term Follow-Up study. Orthop Surg. 2020;12:809–18. 32462816 10.1111/os.12683 PMC7307235

[14] Hernandez NM, Chalmers BP, Perry KI, Berry DJ, Yuan BJ, Abdel MP. Total hip arthroplasty after in situ fixation of minimally displaced femoral Neck fractures in Elderly patients. J Arthroplasty. 2018;33:144–8. 28844629 10.1016/j.arth.2017.07.035

[15] Kellgren JH, Lawrence JS. Radiological assessment of osteo-arthrosis. Ann Rheum Dis. 1957;16:494–502. 13498604 10.1136/ard.16.4.494 PMC1006995

[16] Steinberg ME, Hayken GD, Steinberg DR. A quantitative system for staging avascular necrosis. J Bone Joint Surg Br. 1995;77:34–41. 7822393

[17] Frank T, Osterhoff G, Sprague S et al. The Radiographic Union score for hip (RUSH) identifies Radiographic Nonunion of femoral Neck fractures. Clin Orthop Relat Res 2016. 10.1007/s11999-015-4680-4 PMC4868173 26728521

[18] Garden R. Low-angle fixation in fractures of the femoral Neck. J Bone Joint Surg Br. 1961;43:647–63.

[19] Keller CS, Laros GS. Indications for open reduction of femoral neck fractures. Clin Orthop Relat Res 1980;(152):131–7. 7438595

[20] Leighton RK. Fractures of the neck of the femur. In: Bucholz R, Heckman J, Court-Brown C, editors. Rockwood and Green’s fractures in adults. 6th ed. Philadelphia: Lippincott Williams & Wilkins Co; 2006. pp. 1753–91.

[21] LaVelle D. Fractures and dislocations of the hip. In: Canale S, Beaty J, editors. Campbell’s operative orthopedics. 11th ed. Philadelphia: Mosby Co.; 2008. pp. 3237–308.

[22] Palm H, Gosvig K, Krasheninnikoff M, Jacobsen S, Gebuhr P. A new measurement for posterior tilt predicts reoperation in undisplaced femoral neck fractures: 113 consecutive patients treated by internal fixation and followed for 1 year. Acta Orthop. 2009;80:303–7. 19634021 10.3109/17453670902967281 PMC2823202

[23] Parker MJ, Khan RJK, Crawford J, Pryor GA. Hemiarthroplasty versus internal fixation for displaced intracapsular hip fractures in the the elderly - A randomised trial of 455 patients. J Bone Joint Surgery-British Volume;84:1150–5. 10.1302/0301-620x.84b8.13522 12463661

[24] Cao L, Wang B, Li M, et al. Closed reduction and internal fixation versus total hip arthroplasty for displaced femoral neck fracture. Chin J Traumatol. 2014;17:63–8. 24698572

[25] Leonardsson O, Sernbo I, Carlsson A, Akesson K, Rogmark C. Long-term follow-up of replacement compared with internal fixation for displaced femoral neck fractures: results at ten years in a randomised study of 450 patients. J Bone Joint Surg Br. 2010;92:406–12. 20190313 10.1302/0301-620X.92B3.23036

[26] Do LND, Kruke TM, Foss OA, Basso T. Reoperations and mortality in 383 patients operated with parallel screws for Garden I-II femoral neck fractures with up to ten years follow-up. Injury. 2016;47:2739–42. 27802891 10.1016/j.injury.2016.10.033

[27] Lapidus LJ, Charalampidis A, Rundgren J, Enocson A. Internal fixation of garden I and II femoral neck fractures: posterior tilt did not influence the reoperation rate in 382 consecutive hips followed for a minimum of 5 years. J Orthop Trauma 2013;27:386,90; discussion 390-1. 10.1097/BOT.0b013e318281da6e 23287762

[28] Heetveld MJ, Raaymakers ELFB, van Eck-Smit BL, van Walsun ADP, Luitse JSK. Internal fixation for displaced fractures of the femoral neck. J Bone Joint Surg. 2005 March;87(3):367–73. 10.1302/0301-620x.87b3.15715 15773648

[29] Cullen SE, Sephton B, Malik I, Aldarragi A, Crossdale M, O’Connor M. A Comparative Study of Dynamic Hip Screw Versus Multiple Cannulated Compression Screw Fixation in Undisplaced Intracapsular Neck of Femur Fractures. Cureus 2022 November 17;14(11):e31619. 10.7759/cureus.31619 PMC9759363 36540527

[30] Archibeck MJ, Carothers JT, Tripuraneni KR, White REJ. Total hip arthroplasty after failed internal fixation of proximal femoral fractures. J Arthroplasty. 2013;28:168–71. 22682040 10.1016/j.arth.2012.04.003

[31] Kalsbeek JH, van Donkelaar MF, Krijnen P, Roerdink WH, de Groot R, Schipper IB. What makes fixation of femoral neck fractures fail? A systematic review and meta-analysis of risk factors. Injury. 2023;54:652–60. 36437167 10.1016/j.injury.2022.11.042

[32] Rubio-Avila J, Madden K, Simunovic N, Bhandari M. Tip to apex distance in femoral intertrochanteric fractures: a systematic review. J Orthop Sci. 2013;18:592–8. 23636573 10.1007/s00776-013-0402-5

