# Supplementary material for: The dynamic locking blade plate: seven-year follow-up results of 389 patients with a femoral neck fracture
Source: Eur J Trauma Emerg Surg. 2024 May 31;50(5):2219–27. doi: 10.1007/s00068-024-02552-5 (PMC11599314; doi:10.1007/s00068-024-02552-5)
Supplement: Supplementary file 1 — Supplementary Material 1 [file 68_2024_2552_MOESM1_ESM.docx]

**Supplementary material 1** Independent predictors for avascular necrosis of the femoral head (AVN) of patients with displaced femoral neck fractures (FNF) treated with the Dynamic Locking Blade Plate: uni- and multi-variable Cox regressions.

|  |  | **Univariable analysis** |  | **Multivariable analysis** |  |
| --- | --- | --- | --- | --- | --- |
| **Displaced FNF** |  | *Hazard ratio (95% CI)* | *P-value* | *Hazard ratio* | *P-value* |
| Gender | Male | Reference |  |  |  |
|  | Female | 1.50 (0.72-3.15) | 0.28 | 1.45 (0.64-3.28) | 0.38 |
| Surgeon | Surgeon | Reference |  |  |  |
|  | Surgical resident | 1.30 (0.53-3.19) | 0.57 | 1.44 (0.56-3.74) | 0.45 |
| TAD | ≤25mm | Reference |  |  |  |
|  | >25mm | 1.73 (0.78-3.82) | 0.18 | 1.77 (0.75-4.20) | 0.20 |
| Age | ≤ 65 years | Reference |  |  |  |
|  | 66-75 years | 1.01 (0.44-2.34) | 0.98 | 0.85(0.35-2.10) | 0.72 |
|  | >75 years | 1.44 (0.48-4.30) | 0.51 | 1.79(0.58-5.50) | 0.31 |
| Reduction | Adequate reduction | Reference |  |  |  |
|  | Inadequate reduction | 2.89 (1.27-6.55) | 0.01 | 2.90(1.20-7.01) | 0.02 |
